# Do children with cerebral palsy benefit from computerized working memory training? Study protocol for a randomized controlled trial

**DOI:** 10.1186/1745-6215-15-269

**Published:** 2014-07-07

**Authors:** Gro CC Løhaugen, Harald Beneventi, Guro L Andersen, Cato Sundberg, Heidi Furre Østgård, Ellen Bakkan, Geir Walther, Torstein Vik, Jon Skranes

**Affiliations:** 1 Department of Pediatrics, Sørlandet Hospital, Sykehusveien 1, 4809 Arendal, Norway; 2 Department of Laboratory Medicine, Children’s and Women’s Health, Norwegian University of Science and Technology, Olav Kyrres gate 9, 7489 Trondheim, Norway; 3 Department of Pediatric Habilitation at Østerlide, Stavanger University Hospital, Østerlide PB 8100, 4068, Norway; 4 Cerebral Palsy Register of Norway, Habilitation Center, Vestfold Hospital Trust, Postboks 2168, 3103 Tønsberg, Norway; 5 Department of Child and Adolescent Psychiatry, Vestfold Hospital Trust, Christian Fredriksgate 6., Tønsberg pb. 2325, 3103, Norway

**Keywords:** Cerebral Palsy (CP), Cognitive intervention, Working memory training

## Abstract

**Background:**

Cerebral palsy (CP) is the most common motor disability in childhood (2 to 3 per 1000 live births), and is frequently accompanied by cognitive impairments and behavioural problems. Children with CP are at increased risk of attention deficit disorder with or without hyperactivity (Attention Deficit Disorder (ADD)/Attention Deficit Hyperactivity Disorder (ADHD)) including working memory deficits. The primary aim of this study is to evaluate if cognitive training may improve working memory in children with CP.

**Methods/Designs:**

The study is an investigator-blinded, randomized controlled trial with a stepped-wedge design that will include 115 schoolchildren with CP. Eligible for participation are children with CP, aged 7 to 15 years, who are able to follow instructions and handle a computer mouse. Exclusion criteria are the presence of photosensitive epilepsy, Gross Motor Function Classification System (GMFCS) level V (most severe CP) (Phys Ther 80: 974-985, 2000) and severe visual or hearing impairments. Following assessment of eligibility and baseline cognitive assessment the participants will be randomized to either cognitive working memory training or treatment-as-usual (‘control group’). The intervention is a computer-based working memory training program consisting of 25 daily sessions to be performed over a 5 to 6-week period at home. A neuropsychological assessment will be performed before and 4 to 6 weeks after completed training. When the latter assessment has been completed in the intervention group, the ‘control group’ will start on the same training program. Both groups will meet for a final neuropsychological assessment six months after completed training by an examiner unaware of group adherence.

**Discussion:**

There is limited evidence for the effect of most interventions in children with CP, and evidence is completely lacking for interventions aiming to improve deficits in cognition, learning and behaviour. The proposed multicenter study, will bring forth comprehensive information about cognitive, neuropsychological, and daily-life functioning in children with CP aged between 7 and 15 years. In addition, the study will be the first to evaluate the effects of an intervention method to improve working memory in children with CP. If successful, computer-based working memory training may represent an efficient and cost-effective intervention for this group of children.

**Trial registration:**

ClinicalTrials.gov Identifier: NCT02119364

## Background

Cerebral palsy (CP) is the most common motor disability in childhood (2 to 3 per 1000 live births), and is frequently accompanied by cognitive impairments and behavioural problems [1-4]. Treatment and rehabilitation of patients with CP requires a broad-based and longterm interdisciplinary approach. Children with CP are at increased risk of attention deficit disorder with or without hyperactivity (attention deficit disorder (ADD) and/or attention deficit hyperactivity disorder (ADHD)) [4] and working memory deficits [5]. CP with cognitive deficits represents significant burdens on patients, their families and the society. Even so, evidence-based treatment is lacking for most treatment modalities for children with CP in general, and completely for interventions aiming to improve deficits in cognition, learning and behaviour [6].

The present study is a multicenter controlled clinical trial involving children and researchers from three of five different health regions in Norway, as well as the Norwegian University of Science and Technology (NTNU), and its main research goal will be to evaluate the effects of computer-based cognitive training in children with CP. In addition, this study will be the first to include a comprehensive neuropsychological examination to improve our understanding of cognitive impairments and deficits, as well as cognitive capabilities in children with CP to aid in intervention planning and rehabilitation services.

Cerebral palsy is caused by injuries or developmental anomalies that may involve the cerebral cortex and white matter, deep grey matter nuclei and cerebellum of the developing, immature brain. The brain injury is primarily associated with motor impairments, but also with deficits affecting perception, speech, communication and cognition including deficits in attention and reduced executive functions [3,7-9]. However, children with CP constitute a heterogeneous group and the clinical picture depends on the timing, extent and localization of the brain injury. The two most common subtypes are spastic unilateral and spastic bilateral CP. The unilateral subtype is most common among children born at term, where one main cause is thought to be a vascular catastrophe of the main cerebral arteries (perinatal ‘stroke’) [10]. Approximately half of the stroke cases have their brain damage on the right side, the other half on the left side [8]. Children with unilateral CP have less severe motor impairments and less cognitive problems than those with bilateral CP [11]. Nonetheless, the unilateral group has a significantly increased risk of attention problems, even as pre-school children, as assessed by their parents and teachers as compared to healthy children [4]. Cognitive impairments and problems with attention and executive functions are more common among children with CP than among typically developing children [12-16]. Among preterm born children, both with and without CP, cognitive impairments including reduced working memory and high prevalence of ADHD persist into adulthood [17-19].

Working memory represents a central component of attention and executive functions and may be defined as the ability to keep information ‘online’ and at the same time manipulate the information. Working memory is regarded a contemporary storage system that is under attention control [20] and supports our ability for goal-directed behavior and other higher order cognitive processes required in situations that are novel [20]. It is also considered to be a prerequisite for other executive functions, such as reasoning and planning, and for predicting intelligence and academic success [21,22]. Functional brain imaging has shown that working memory is primarily supported by a neuronal network in the frontal and parietal lobes [23] and that increased activity in these areas of the brain correlates positively with measures of working memory capacity [24]. Executive and attention functions describe the ability to self-regulate behaviour, cognition and emotions [25,26]. These functions are primarily localized in the frontal part of the brain. Since the frontal lobe of the brain is connected to other brain areas through fronto-parietal tracts as well as long association tracts, brain injuries in parts of the brain other than the frontal lobe that affect connectivity may also lead to symptoms of frontal lobe impairment [27]. Both we [4,18,28,29] and other authors [30-32] have reported that children born preterm and children with CP have an increased prevalence of symptoms of reduced attention and problems with working memory similar to the problems found in children with ADD and/or ADHD.

There is also a lack of studies examining how cognitive deficits may differ between children with various CP subtypes and within the same subtype; for example in children with the spastic unilateral CP subtype, between those with an injury on the right and left side of the brain, respectively. In adults suffering from stroke it is unclear whether a right or left side brain injury affects working memory differently [33]. In a recent study by Laures-Gore *et al*. [33] stroke patients with a left brain injury had more impaired verbal working memory than those with an injury on the right side, consistent with an early report by Black and Strub [34]. However, other studies have not found any differences in working memory between right and left side brain damage after stroke in adults [35]. In children with CP this has, to the best of our knowledge, not been studied despite the higher prevalence of attention problems. The extent of more specific problems, such as with working memory, may depend on CP subtype and be related to perinatal complications [36]. The spastic bilateral subtype that mainly involves the lower extremities (known as diplegic CP) is particularly common in children born preterm [10]. In preterm children, tracts and networks of neural connections in the brain are particularly susceptible to damage due to reduced perfusion and oxygen supply to the periventricular water-shed areas in deep white matter [37]. Injuries in these areas, where projectional cortico-spinal motor tracts, especially to the lower extremities, are passing through and may be damaged, explain the motor impairment. However, such diffuse or focal white matter injuries may also lead to problems of perception, cognition, attention and other higher brain functions (executive function) caused by damage to commissural and association tracts with compromised connectivity and networking [7]. Few studies have assessed attention and executive functions in preterm born children with CP but one study reports that children with spastic diplegic CP do have problems with inhibition control likely caused by deep white matter damage [14]. Learning difficulties and problems with social functioning have also been described in children with spastic diplegic or hemiplegic (affecting one side of he body) CP due to impaired attention and executive functions [25]. In young adults born very preterm with a birth weight of less than 1500 grams, a study by Løhaugen *et al.* indicated that working memory may be especially impaired in those with CP, even into adulthood [18].

### Training of working memory

Until recently it was believed that working memory could not be influenced by stimulation or training. However more recent research, including our own at NTNU, has shown that working memory capacity can be improved through cognitive training [16,38-40]. A computer-based program (Cogmed Robomemo™, http://www.pearsonassessment.com) for the training of working memory of children with ADHD was developed by Professor Torkel Klingberg at the Karolinska Institute in Stockholm in 1999, and the first training results were published in 2002 [38]. The program improved working memory in the trained tasks; however, tasks not trained in the program also improved. This indicated a generalization effect to tasks such as understanding room-direction and complex problem solving [38]. The findings were confirmed in a randomized controlled trial of children with ADHD showing that Cogmed Robomemo™ training improved both verbal and visual working memory and that the parents reported fewer symptoms of inattention in their children after completion of the training [41]. These studies were, however, limited to participants without general learning difficulties or cognitive impairments. In the first Robomemo study that included adolescents born with an extremely low birth weight (birth weight of less than 1000 grams), Løhaugen *et al.*[42], included participants with intellectual impairments and two participants with CP (17). They found that even when these participants were included the Cogmed Robomemo™ intervention improved working memory. The positive effect was observed on trained as well as untrained tasks, and also on memory and reduced symptoms of ADHD [16]. Other studies have shown that children without ADHD but with difficulties with working memory, can improve their working memory with training [43]. These findings suggest that training of working memory may have significant impact on academic performance.

### How does training affect the brain and what are the possible biological mechanisms?

Studies have shown that training of working memory also induces neurobiological changes such as increased activity in the prefrontal cortex and the parietal lobe [44]. These areas are associated with response inhibition and the ability to reason [24]. Overlapping neural systems could be a reasonable explanation of the generalized effects of working memory training to other cognitive functions. Furthermore, training of working memory has been shown to increase the density of dopamine receptors in the prefrontal cortex [45] and has also been associated with structural changes in the white matter of the brain [46]. This is in support of a training-induced plasticity of the neural networks underlying working memory, response inhibition and the ability to reason. If working memory and executive functioning can be improved by training it would be reasonable to assume that training will also have an effect on the activities in daily life that are dependant on working memory. A third mechanism constitutes changes in white matter microstructure. Working memory capacity is associated with the integrity of the white matter network between the parietal and the frontal lobes [47]. Takeuchi *et al.* found higher fractional anisotropy (FA) values in parietal white matter tracts parallel to corpus callosum after working memory training in healthy elderly adults, indicating improved microstructure [46]. The degree of increase in FA depended on the magnitude of training. This may be explained by improved myelination leading to an increased efficiency of signal transfer between the different parts of the brain. Increased cortical thickness in healthy controls after memory training has also been reported [48].

## Methods/Design

### Objectives

The main goals of the present study are as follows: To present cognitive profiles reflecting neuropsychological functions in children with different subtypes of CP with varied aetiology. Also, to study of a computer-based training program can improve the working memory capacity of children with CP (near transfer effect), and improve learning abilities and behaviour. While learning abilities are assessed by neuropsychological learning and memory tests, behaviour will be assessed by questionnaires (the Vineland Adaptive Behaviour Scale and ADHD rating scale) given to parents and school teachers (far transfer effect). If the intervention is effective, will the beneficial effects persist over time? Assessment will include questionnaires and neuropsychological tests at six months post-training.

To present cognitive profiles reflecting neuropsychological functions in children with different subtypes of CP with varied aetiology.

To study if a computer-based training program can:

Improve the working memory capacity of children with CP (near transfer effect).

Improve learning abilities and behaviour. While learning abilities are assessed by neuropsychological learning and memory tests, behaviour will be assessed by questionnaires (the Vineland Adaptive Behaviour Scale and ADHD rating scale) given to parents and school teachers (far transfer effect).

If the intervention is effective, will the beneficial effects persist over time? Assessment will include questionnaires and neuropsychological tests at six months post-training.

This study will provide new knowledge on the effectiveness of a computer-based cognitive training program in improving the daily-life function and behavioral problems of children with CP. We hypothesize that neuropsychological deficits and impairment will be frequent in this group. Our main hypotheses are that working memory can be improved through cognitive training in children with bilateral as well as unilateral CP, the two most common CP subtypes. We also hypothesize that improved working memory will result in improved learning abilities and less symptoms of inattention.

### Design

**Figure 1:**
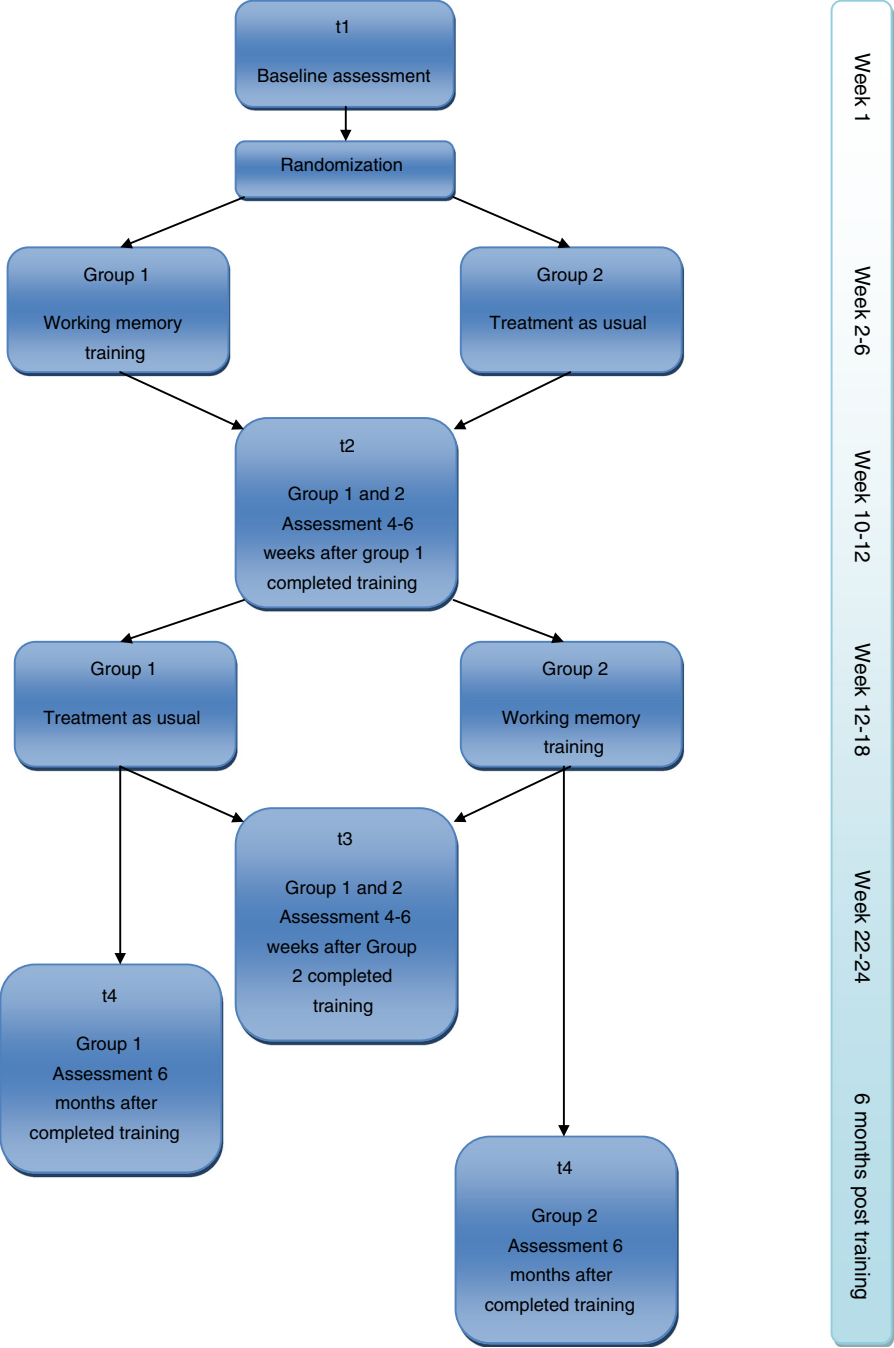
Study design.

The study is a multicentre, clinical controlled trial applying a stepped-wedge design, as described by Brown and Lilford [49]. The children will be examined at baseline (before the start of training - t1) and immediately after completed training (after four to six weeks - t2). According to the stepped-wedge design, group 1 will start training while the other group will wait and serve as the control. Group 2 will then start training when group 1 has completed its training (Figure 1). This design ensures a well matched control group enabling adjustment for the natural cognitive development that occurs over time. Group 1, the first to receive the training, will also meet for the follow up after group 2 has finished the training to ensure that the researchers remain blinded to group adherence throughout the study (t3). All participants will meet for a longterm follow-up six months after completing training for each group respectively (t4). The primary endpoint will be the assessment performed at four to six weeks after training (t2 and t3).

### Participants

Children aged between 7 and 15 years with CP will be invited to participate. Through the Cerebral Palsy Register of Norway (CPRN) we have identified 70 children with unilateral CP and 45 children with bilateral CP born in these health regions. The children must be able to use a personal computer mouse and they will be included if they understand the instructions and the intention of the computer game, regardless of IQ. Children with Gross Motor Function Classification System (GMFCS) level V (most severe CP) [1] or who have severe visual or hearing impairments, or photosensitive epilepsy will be excluded. Participants will be recruited from the following counties: Nord- and Sør-Trøndelag (Health region Mid-Norway), Vestfold, Telemark, Aust- and Vest-Agder (Health region Southeast Norway) and from Rogaland (Health region West Norway).

### Outcome measures and assessment procedure

The primary outcome measure is the spatial span task from the Wechsler Memory Scale 3rd edition, assessing spatial working memory at four to six weeks after training (t2/t3) (Table 1).

**Table 1:** Neuropsychological tests and parental questionnaires at baseline and follow-up assessments

			**Time point**			
**Function assessed**	**Test battery**	**Subtests**	**T1**	**T2**	**T3**	**T4**
General cognitive ability	WISC-IV	Subtests included in Full IQ	X			
Attention	NEPSY II	Animal sorting	X	X	X	X
		Inhibition	X	X	X	X
		Auditory attention and response set	X	X	X	X
Executive	NEPSY II	Verbal fluency	X			
Social perception	NEPSY II	Affect recognition	X	X	X	X
Working memory	WISC-IV	Digit span	X	X	X	X
		Letter-number sequencing	X			
		Arithmetic	X	X	X	X
	WMS-III	Spatial span board	X	X	X	X
Language	NEPSY	Phonological processing	X	X	X	X
Learning/memory	NEPSY II	Memory for designs	X	X	X	X
		Memory for designs delayed	X	X	X	X
		Word list interference	X	X	X	X
		List memory	X	X	X	X
		List memory delayed	X			
		Narrative memory	X			
Visual-spatial processing	NEPSY II	Geometrical puzzles	X			
Questionnaires	BRIEF	Behavior Rating Inventory For Executive Functions	X	X	X	X
	VABS	Vineland Adaptive Behavior Scale	X		X	X
	ADHD RS	Attention Deficit Hyperactivity Disorder Rating Scale	X	X	X	X
Socioeconomic status of parents	SES	Hollingshead two factor index of social position that is based on the parent’s education and occupation	X			

Developmental NEuroPSYchological Assessment 2nd edition; IQ: Intelligence quotient; NEPSY II; SES: socio-economic status; T1: Baseline assessment; T2: Assessment after training; T3: longterm follow-up at six months post-training; WISC-IV: Wechsler Intelligence Scale for children 3rd edition.

### Neuropsychological assessment at baseline (t1)

The examination at baseline will include a full WISC- IV. Age-appropriate norms from the Scandinavian standardization will be used to calculate IQ scores. The comprehensive ‘Developmental Neuropsychological Assessment’ (NEPSY) will be applied to study memory and learning, language, attention and executive functions. At baseline the assessment lasts for approximately 3.5 hours including breaks. After completion of the baseline assessment, participants will be randomized to either training or treatment-as-usual.

### Neuropsychological assessment at follow-up (t2/t3 and t4)

To study the short-term and longterm effects of training on working memory, a training index is provided by Cogmed (http://www.pearsonassessment.com) based on the individuals training results in the computer program (’trained tasks’) [16]. The effects of the training on non-trained working memory tasks will be assessed with the following standardized neuropsychological tests: spatial span board (WMS-III), letter-number sequencing and digit span (WISC-IV). Subtests from the NEPSY battery will also be included. At both the short-term (t2) and longterm (t3) follow-up the same battery of neuropsychological tests will be administered, lasting for approximately 1.5 hours including one break.

### Questionnaires

Parents will be asked to complete questionnaires regarding their child’s functioning in daily life (measured using the Vineland Adaptive Behaviour Scale) at baseline and at the six-month follow-up. Symptoms of attention deficit and hyperactivity (measured using the ADHD rating scale) as well as executive functions (measured using the Behaviour Rating Inventory of Executive function - BRIEF) will be administered at all time points (t1-t3). The child’s main teacher will be asked to complete the ADHD rating scale, as well as the BRIEF-teacher rating at baseline and at four to six-weeks after completing the training.

### Intervention

The Cogmed Robomemo™ program is designed as a computer game in which an animated robot gives different tasks to be solved by the child. During each training session the child is presented with eight different tasks. The program is adaptive in that its difficulty level increases or decreases depending on the individual child’s performance in any given task. If the child makes consecutive errors on a task the program will decrease the load of the task by reducing the number of items presented to be held in working memory. If the child successfully solves consecutive tasks the number of items presented will increase. The child will do the training at home using his or her own computer for about 30 to 45 minutes each day, five days a week for five weeks. A certified coach follows the training progress through a secure website and supervises the child by contacting the family once a week to give feedback, as well as encouragement and advice for further improvement of the results. All participating sites have certified coaches trained by Cogmed to perform this task. The coaching and assessments will be performed by other researchers to enable assessors to be blinded to group adherence (training versus no training group).

### Statistical methods

Data will be analysed using the IBM SPSS statistics software for Windows, version 19 (IBM Corp. Released 2010. IBM SPSS Statistics for Windows, Version 19.0. Armonk, NY: IBM Corp, USA) (Illinois, United States). Differences in group means for variables with a normal distribution will be compared with the Student’s *t-*test. Outcome measures not normally distributed will be analyzed using the Mann-Whitney *U* test. The Wilcoxon signed-rank test for two related samples will be used to compare scores at the two time points (baseline versus immediately after training). A univariate general linear model will be used to compute mean values for the neuropsychological tests and questionnaires adjusted for gender and SES, with the group as the fixed factor and sex and the SES as covariates. A full data analysis plan will be drafted before unmasking group adherence.

### Estimation of sample size

The aim of the present study is to demonstrate a clinically significant effect of the training on working memory, and we defined this as a 10% score increase on the main outcome measure, the spatial span from the WMS-III, in those children who had performed the training as compared with those in the control group. In order to estimate the sample size needed to meet this aim, we applied results (mean and SD) from one of our previous intervention studies in adolescents born with extremely low birth weights (ELBW; birth weight (BW) of less than 1000 g), of whom two participants had CP (Løhaugen *et al.*[18]). Using these figures, we estimated (sample power 3) that we would need to recruit 100 participants in order to show a 10% improvement in spatial span following training, with an 80% power and a two-tailed alpha of 0.05. In order to allow for a dropout rate of 15%, we decided to include 115 participants.

### Ethical perspectives

The Regional Committee for Medical Research Ethics (Norwegian Health Region II) approved the study protocol (project number: 2012/298). All invited participants consented to be registered and to receive information about relevant research studies through The Cerebral Palsy Register of Norway (CPRN). Information including consent forms was sent to the families by mail from the CPRN. The consent forms are to be returned to the CPRN to be transferred to the principal investigator of the research study. The families will then be contacted to schedule an appointment for baseline assessment. If the cognitive and neuropsychological testing indicates learning disorders, the child will be offered a referral to appropriate institutions to receive further assessment and diagnostic evaluation. All parents will be offered a meeting with the neuropsychologist to discuss the child’s results if wanted, as well as written reports. The CONSORT statement and the CONSORT statement for non-pharmacological interventions will be followed in the reporting of results [50,51].

### Dissemination plan

The following publications are planned:

Based on results from examinations before training:

Paper 1: Cognitive profile (IQ) in Norwegian children with CP born preterm or at term.

Paper 2: Neuropsychological function in Norwegian children with cerebral palsy.

Based on results from after the training of working memory:

Paper 3: Short- and longterm effects on cognitive function of a computer-based working memory training program in children with CP.

Paper 4: Effects of a computer-based working memory training program on activities of daily living and psychological wellbeing in children with CP.

Based on results from examinations before training: Paper 1: Cognitive profile (IQ) in Norwegian children with CP born preterm or at term. Paper 2: Neuropsychological function in Norwegian children with cerebral palsy. Based on results from after the training of working memory: Paper 3: Short- and longterm effects on cognitive function of a computer-based working memory training program in children with CP. Paper 4: Effects of a computer-based working memory training program on activities of daily living and psychological wellbeing in children with CP.

## Discussion

It has been reported that the majority of children (80%) with a working memory capacity below the 10th percentile experience major problems in reading and writing as well as in mathematics [43]. In children with CP, reduced working memory capacity is prevalent [36], and has been shown to be related to deficits in arithmetic performance [52]. A study by one of the researchers behind the present proposal described reduced working memory in children with learning difficulties related to reading [53]. Other studies have found working memory to be relatively independent of a child’s general level of intelligence [54], and to be a better predictor of academic success than IQ [55]. Thus, taken together, all these studies suggest that improving working memory is likely to have a number of general positive effects on other cognitive functions, academic performance and behaviour. We therefore want to examine whether this also is true for children with CP.

The intervention method tested here has components that make it user friendly and cost-effective regarding time and economic resources in that it is computer-based, performed at home and has a relative short duration of intervention. If this kind of cognitive training proves to be effective in children with CP, we have provided important information and knowledge in the further development of cognitive intervention methods that may be applied within the child rehabilitation system.

Children with CP are often in need of multidisciplinary rehabilitation and special education interventions. There is a lack of evidence-based knowledge regarding cognitive function and effects of intervention methods in this patient group. In addition, assessing a comprehensive cognitive profile in children with CP will provide a better knowledge base for the planning of treatment and rehabilitation. To avoid ineffective and unnecessary treatment at high costs more scientific studies are needed. Improving working memory may have positive consequences at both an individual and family level, reduce the need for special education and improve social and daily-life functioning. The proposed study will enable recommendations regarding the effect of cognitive training in children with CP.

## Trial status

Recruitment startet in August 2013 and will be continuing until June 2015. Currently recruiting.

## Competing interests

The authors declare that they have no competing interests.

## Authors’ contributions

GCC L: Conceptualizing the study, writing and revising the research protocol. HB: Conceptualizing the study, writing and revising the research protocol. GA: Conceptualizing the study, writing and revising the research protocol. CS: Planning the study, revising the research protocol, performing coaching. HFØ: Conceptualizing the study, writing and revising the research protocol. EB: Planning the study, revising the research protocol, performing coaching. GW: Conceptualizing the study, revising the research protocol, performing coaching. TV: Conceptualizing the study, writing and revising the research protocol. JS: Conceptualizing the study, writing and revising the research protocol. All authors read and approved the final manuscript.
